# Diversity of Vaginal Microbiome in Pregnancy: Deciphering the Obscurity

**DOI:** 10.3389/fpubh.2020.00326

**Published:** 2020-07-24

**Authors:** Parakriti Gupta, Mini P. Singh, Kapil Goyal

**Affiliations:** Department of Virology, Post Graduate Institute of Medical Education and Research (PGIMER), Chandigarh, India

**Keywords:** microbiome, *Lactobacilli*, pregnancy, virome, preterm labor

## Abstract

Human microbiota plays an indispensable role in physiology, nutrition and most significantly, in imparting immunity. The role of microbiota has remained cryptic for years, until recently meticulous studies revealed the interaction and dynamics of these microbial communities. This diversified state is governed by hormonal, behavioral and physio-chemical changes in the genital tract. Many inclusive studies have revealed “*Lactobacillus*” to be the most dominant member of vaginal flora in most of the healthy, reproductive age group and pregnant females. A total of five community state types have been described, out of which four are dominated by *Lactobacillus* while the fifth one by facultative or strict anaerobic species. A variation between species stability and gestational age has also been revealed. Studies have divulged a significant higher stability of vaginal microbiota in early stages of pregnancy and the same increased subsequently. Inter-species and racial variation has shown women belonging to White, Asian, and Caucasian race to harbor more of the anaerobic flora. The vaginal microbiome in pregnancy play a significant role in preterm and spontaneous labor. This *Lactobacillus*-rich microbiome falls tremendously, becoming more diverse in post-partum period. Apart from these known bacterial communities in human vagina, other microbial communities have also been traced. The major fragment is constituted by vaginal viral virome and very little information exists in relation to vaginal mycobiome. Studies have revealed the abundance of ds DNA viruses in vaginal microbiome, followed by ssDNA, and few unidentified viruses. The eukaryotic viruses detected were very few, with *Herpesvirales*, and *Papillomaviridae* being the only pathogenic ones. This flora is transmitted to infants either via maternal gut, vagina or breast milk. Recent studies have given an insight for vaginal microbiome, dissociating the old concept of “healthy” and “diseased.” However, more extensive studies are required to study evolution of virome and mycobiome in relation to their association with bacterial communities; to establish and decode full array of vaginal virome under the influence of genotypic and environmental factors, using novel bioinformatic, multi-omic, statistical model, and CRISPR/Cas approaches.

## Introduction

Human microbiota is an array of microbes, including archaea, protista, bacteria, fungi, and viruses that exist on and within the human tissues, fluids and body cavities, along with varied anatomical sites; whereas microbiome is an aggregate of the genetic materials of resident microbiota (1, 2). This microbiota plays an indispensable role in human physiology, nutrition, and most significantly in imparting immunity. The microbial communities exemplify a segment of first-line defense of human body by competitive exclusion of the invading microorganisms. A significant number of microorganisms live in a mutualistic, harmonious relationship in human body, whilst a few of them become opportunistic in immunosuppressed conditions, leading to acute, fatal, and chronic conditions. Notwithstanding the role of microbiota in human physiology, the minutiae remain cryptic for years until recently meticulous studies revealed the configuration, function, interaction, and dynamics of these microbial communities amongst each other and with the human host, which enable different individuals to behave differently when encountered with external stress. The major landmark in this came with the human microbiome project (HMP; 2008–2013), with an aim to profile the microbial composition of healthy adults, 18–40 years old, with special interest to know the impact of human microbiota in disease and health, taking care that the microbiome included in study is minimally perturbed. The core microbiome in this project was studied from five anatomical sites viz skin, nasal passages, oral cavity, gastrointestinal tract and urogenital tract and the total number of microbiomes interrogated were 3,000 from 300 healthy individuals. The analytic tools used in HMP included taxonomic profiling by 16SrRNA sequencing and more comprehensive analysis by metagenomic sequencing (1, 3, 4) and it revealed the taxonomic composition of human microbiome. However, it was noted that this could not serve as a sole correlate with host phenotype, (5) hence it acted as the underpinning for the second phase of this project; Integrative HMP (iHMP). It has been delineated that altered microbiota can act as an initiating factor for many disorders like obesity, diabetes, inflammatory bowel disease, bacterial vaginosis (BV), preterm labor (PTL), and many others (2). The iHMP was devised to reconnoiter the same host-microbiome dynamics over a period of time. In this second phase, the multi-omics approach was used to target the specific disease conditions to have a broader perspective of microbiome interactions in health and disease, with known modulations in specified states like preterm labor in pregnancy, inflammatory bowel disease and pre-diabetes (6). All these three studies have finished the first phases of their research recently (7–9). Apart from HMP, many other studies have shown the influence of microbiota in different dysbiotic and diseased states. The maximum association between health and disease has been related to the gut. The gut microbiome is the vastest of all, encompassing different generas and phylas contributing to the dynamic human physiology and immunity (10). As the elements of microbiome were unveiled, research on microbiota of sites other than those included in HMP, like urinary tract, vagina, respiratory tract, central nervous system, conjunctiva, began (11–16). Of these, vaginal microbial communities represent a fine, balanced, mutualistic association between microorganisms, and human physiology. The bacterial communities derive nutrition from the sloughed cells, glandular secretions of the host to replenish their continuously dwindling counts, while these help the host by preventing colonization by non-resident, invading microbes (17, 18). The traits of this dynamic, co-evolutionary vaginal ecosystem have remained cryptic for years till the advent of comprehensive, culture-independent approaches. An interesting correlation between Vitamin D, PTL, and vaginal microbiome has also been reported.

## Vaginal Microbiota

**Figure 1:**
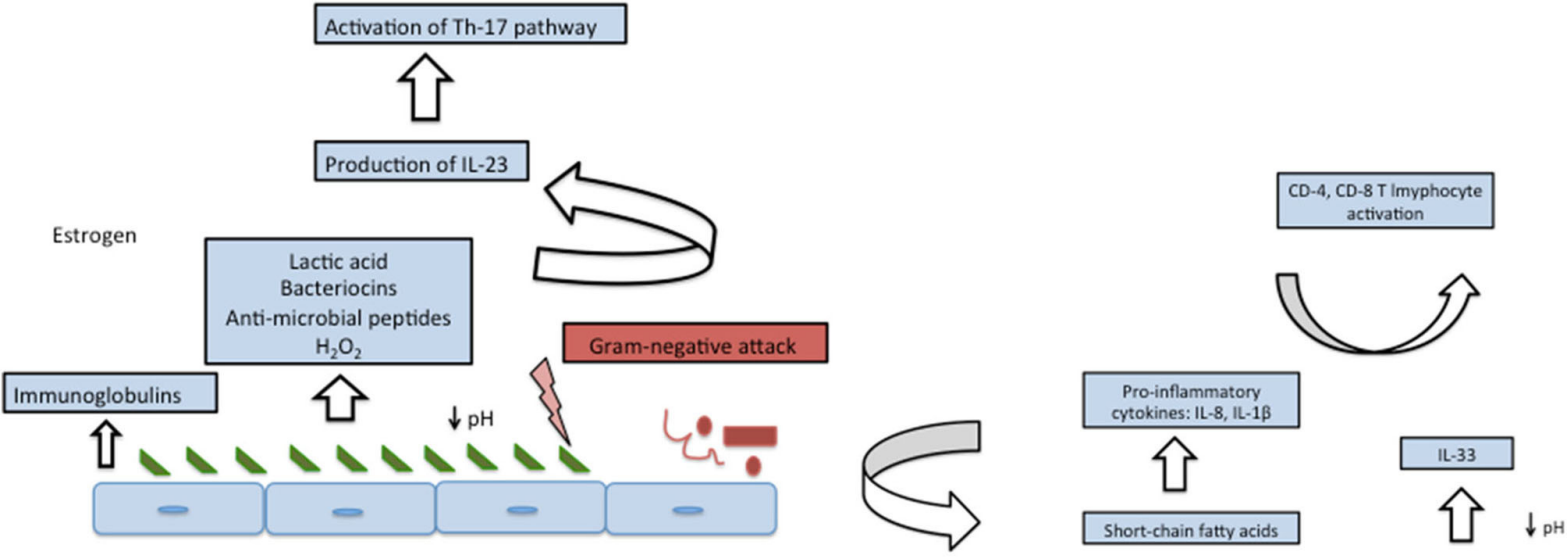
Vaginal homeostasis and microbiome (left panel) and fluctuations after encountering gram-negative attack (right panel).

**Figure 2:**
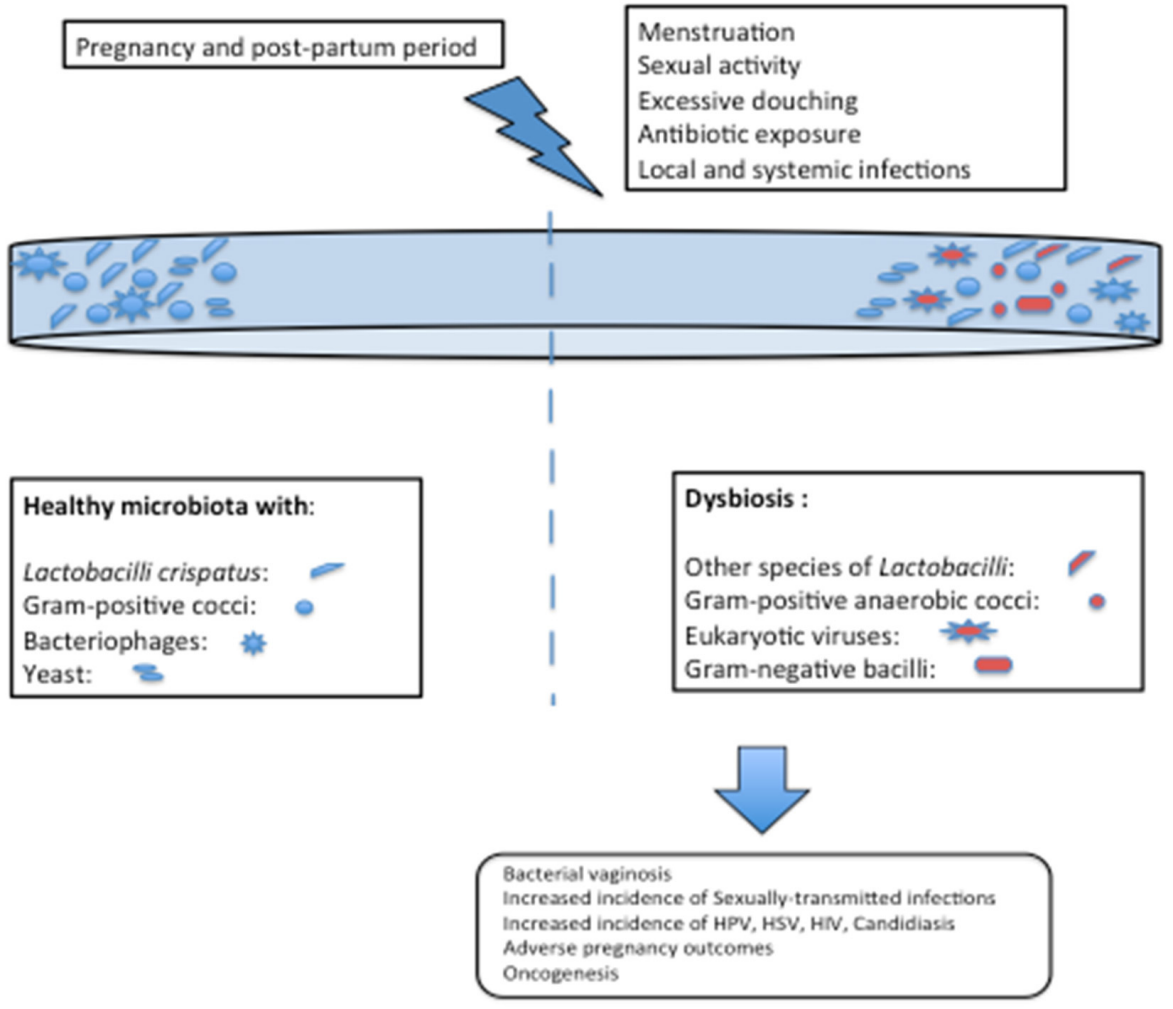
Risk factors for alteration in vaginal microbiome and consequences of dysbiosis.

Many inclusive studies, both culture-dependent and culture-independent have revealed “*Lactobacillus”* to be the most dominant member of vaginal flora in most of the healthy, reproductive age-group females. Primarily, it was assumed that this notion might be skewed due to the availability of only culture-dependent techniques that disregards the growth of fastidious organisms but recently, analysis based on 16SrRNA and sequencing have revealed the same results (19–21). *Lactobacilli* are very well-adapted in vaginal environment and benefit the host by virtue of production of lactic acid as their fermentation by-product, that lowers the pH of vagina to ~3.5 (22). Such a low pH is inhibitory to many other invading microbes, thereby imparting a protective role. Apart from the lactic acid, *Lactobacilli* also generate bacteriocins (23, 24), that are bactericidal, proteinaceous compounds with a very narrow-spectrum of killing which is achieved by enhancing the permeability of the target cell membrane. Hydrogen peroxide, H_2_O_2_, is another defensive factor produced by *Lactobacillus*. The role of H_2_O_2_ is controversial as its production is enhanced in aerobic conditions, whereas the vaginal environment is usually anaerobic. Moreover, one of the major species *L. iners* does not produce H_2_O_2_, thereby further casting further doubt on its significance. Since the details are yet unknown, it is possible that H_2_O_2_ might act as a surrogate marker for some cryptic biological marker (25, 26). Apart from the by-products produced by *Lactobacilli*, it is interesting to note that *Lactobacilli*-enriched vagina, when encountered with any gram-negative attack, imparts a species specific stimulatory effect on our innate immune system by enhancing the production of IL-23, which preferentially activates Th-17 pathway (27, 28). Figures 1, 2 depict the homeostasis and dysbiosis in vaginal microbiome.

## Species Variation of *Lactobacillus*

The most common resident *Lactobacilli* of human vagina include *L. crispatus, L. iners, L. gasseri*, and *L. jensenii*. Prior to culture-independent techniques, *L.crispatus* was considered as the predominant species (29), but after the advent of culture-independent methods, *L.iners* has been found to be the predominant member (30–32). According to a recent study, *L.iners* has been noted in the vaginal flora of 83.5% of the subjects and as dominant member in 34%. The prevalence of *L. crispatus* was 64.5% in all the subjects with dominance in another 26.2%*, L. gasseri* in 42.9% of subjects with dominance in another 6.3% and *L. jensenii* in 48.2% with dominance in 5.3% (32). Another significant feature was the association of different *Lactobacillus* species with varied pH. *L. crispatus* transcends to lower the vaginal pH upto ~4, while other species have capability to impart a comparatively higher pH of ~4.5–5 (32).

It has been shown by many studies that *L. crispatus* has a strong association with elimination of *Gardenella*, exhibiting a much stable microbiome. On the contrary, *L. iners* have been seen to co-exist with *Gardenella*, providing a less stable microbiome and might be implicated in pre-term labor (PTL) by supporting the existence of other harmful species or by other cryptic mechanisms (33–35). These findings are in contradiction to the study by Kindinger et al. (36) who noted no association between *L. iners* and *Gardenella*. The differences might be due to the varied sequencing and techniques used; V1–V3 primers in one study while while 16S rRNA sequencing in the other. A total of five community state types (CST) have been described, out of which four (I, II, III, and V) were dominated by *Lactobacillus*, while the fourth one was noted to be dictated by facultative or strict anaerobic species (32). Contrary to the common belief, 20–30% of the otherwise, healthy females were found to lack this genus fully and were seen to harbor a sundry array of strict or facultative, anaerobic bacteria. This was associated with somewhat higher vaginal pH of ~4.7–6.5 (31, 32, 37) and this high pH predisposes these women to other genital infections (22, 38).

The other genera residing the vagina include *Corynebacterium, Atopobium, Gardnerella, Mobiluncus, Peptoniphilus, Anaerococcus, Prevotella, Finegoldia, Sneathia*, Lachnospiraceae taxon *Lachnovaginosum genomospecies* (BVAB1), and *Eggerthella* (31, 32, 37, 39–46) Other under-recognized members are *Streptococcus, Atopobium, Megasphaera, Leptotrichia*, and *Straphylococcus*. All these organisms have the capability to reside in such a low pH due to the utilization of lactic acid as these are either homolactic or heterolactic fermenters. Apart from the intrinsic pH, other factors such as pregnancy, menstruation, dysbiosis, use of oral contraceptives, lubricants, antibiotics, and douching also play a role in sustainability of these microbial communities (47–49).

## Racial Differences in Vaginal Microbiota

Inter-species and racial variation in microbiota has also been noted. Hispanic and black women have been reported to harbor more of the anaerobic flora, than *Lactobacillus* with dominant CST IV, while most of the Asian and Caucasian race have *Lactobacillus* as the dominant member. Multiple species of *Lactobacillus* have been isolated from these women, but females of black race had more of the single-species dominant cluster, while multiple species of *Lactobacilli* in single female were isolated from Caucasian women (31, 32, 37, 50). Studies have shown a strong association of *L. crispatus* with lower probability of PTL in Caucasian population, while a weaker or no association exists in African population (33, 36). Moreover, inconsistent results have been noted for *L. iners, Gardenella* and PTL, demanding need of an in-depth study for the same. Recent study conducted by Serrano et al. (51) in 300 European, African and Hispanic females each, has revealed a lower prevalence of *G. vaginalis, A. vaginae, Prevotella* cluster 2, *P. bivia*, and *S. amnii* amongst African women, while in Hispanic females, a significantly higher abundance of *A. vaginae* and *P. bivia* was reported. Pregnant women of European lineage showed only a lower prevalence of *L. crispatus* accompanied by a richer prevalence of *L. iners* (51). In all these females, the microbiome shifted more toward a stable, *Lactobacillus*-dominant profile with a major shift in early stages of pregnancy, significantly in women of African or Hispanic lineage. Further studies are required to understand the effect of both genomic and environmental factors in the changing vaginal microbiota. A recent analysis of cervicovaginal microbiota of 2,000 pregnant females has revealed significant association with spontaneous labor, more so in African-American lineage. The utmost astounding aftermath of the study was inverse association of β-defensin 2 with spontaneous preterm labor, but was ethnicity-determined. The risk of spontaneous labor increases in African-Americans with low levels of β-defensin 2, while the reverse is true for non-African Americans (52).

Apart from the racial diversity, vaginal microbiome also varies depending on the mode of delivery, sexual partner (53), health, diabetes status of the mother (54), and between monozygotic and dizygotic twins (55). Vaginal delivery has been illustrated to be favorable for microbiome of the infant, as established by baptism theory (56, 57).

## Vaginal Microbiota in Health and Disease

Since the mere absence of *Lactobacilli* and mere presence of gram-negative organisms does not correlate with the disease, there is an urgent need to modify the previous “Amsel” and “Nugent” scoring system, which consider the presence of *Lactobacilli* as a marker for healthy vagina and vice-versa. The implementation of these criteria which merely use *Lactobacilli* as a marker of healthy vagina, explains the highly reported incidence of bacterial vaginosis (BV), a dysbiotic state of vaginal microflora; high rate of treatment failure and recurrence (41, 58). Recently, BV associated bacteria (BVAB), belonging to *Firmicutes* and *Actinobacteria*, have also been identified, that can be implicated in such dysbiotic states. Moreover, the reliability of such criteria is challenged more in pregnancy where altered pH due to the excessive vaginal discharge alters the microbiota (58, 59).

Daily changes in the dynamic composition of vaginal microbiota have been noted in many studies (60–63). Both types of communities exist, the ones changing significantly over shorter spans, and those that remain constant and stable over a longer duration of time. Maximum changes are seen to occur during menstruation, followed by sexual activity, however a relatively stable microflora is seen at times of high estrogen and progesterone levels (64).

## Vaginal Microbiota in Pregnancy

Pregnancy is a state of diverse physiological changes to adapt fetus to human body and vice-versa. This diversified state is governed by hormonal changes leading to immune modulation, behavioral changes, physio-chemical changes in the mucosa, and changes in the genital tract (65–67). All these factors further drive the modulation in structure and function of microbiome, making it unique from non-pregnant females. Only few studies have been conducted to know the microbiome of pregnant females, as most of the earlier studies have focused on a non-pregnant vagina (68–72). Recently, a study conducted by Freitas et al. (73) using sequencing of *cpn*60, a universal target and 16S rRNA resolution, has shown significant variations in microbiome of pregnant and non-pregnant females. The microbiota in pregnant vagina is less rich and less diverse as compared to non-pregnant vagina, with a predominance of *Lactobacillus* species. This microbiome was characterized in 182 pregnant females of 11–16 weeks gestation and 310 non-pregnant females, via pyrosequencing of *cpn*60 region and revealed six different community state types profiles (73). Aagaard et al. (4) have revealed the presence of microbiota at three different zones within the vagina of 24 otherwise-healthy pregnant females and noted that there was no site-specificity of any kind. The authors showed that the pregnant vagina was less rich and less diverse than the non-pregnant vagina (68). Antonio et al. (70) have also reported lesser diversity and richer *Lactobacilli* flora in vagina of 12 pregnant females. Additionally, a variation between species stability and gestational age has also been revealed. The authors found a significant higher stability of vaginal microbiota in early stages of pregnancy and the same increased with gestational age (69). The etiology behind these differences is still not well-established but it has been allied to sex hormone levels, with estrogen increasing the thickness of vaginal mucosa, thereby increasing the glycogen deposition. This glycogen deposition acts as a chemotactic agent for microbes as this is major substrate used by these microbes, which is broken to glucose and then fermented to lactic acid, thereby playing a significant role in lowering the vaginal pH (60, 74, 75). The estrogen level is low in early childhood, when diphtheroids and enteric organisms predominate, and again falls significantly after menopause, increasing the colonization by enteric pathogens due to absence of glycogen and glucose in vagina.

## Role of Microbiota in Adverse Pregnancy Outcome

The available information about the vaginal microbiome incited the need to find any association of this varied microbiome in pregnancy with adverse pregnancy outcomes. The vaginal microbiome in pregnancy have been shown to play a significant role in PTL and spontaneous labor. PTL is defined as the delivery of a baby before 37 weeks of gestation, that complicates ~15–30% of pregnancies worldwide and is a major cause of neonatal morbidity and mortality. It remains one of the significant and unsolved problems for obstetricians due its multifactorial etiology. Though the mortality arising from PTL has reduced in the recent past, but the incidence is still static (76). The major contributing factors include premature rupture of membranes, intra-amniotic infections, ascending infections, cervical insufficiency, stress, vascular disorders etc. Ascending genital infections alter the delicate materno-fetal immune balance by release of toxins and array of enzymes compromising the fetal membrane cover and subsequently, disrupting the membranes (77–79). This genital microbial flora can either be endogenous or acquired during an infectious process. Most of these infections are occult accounting for ~25% of PTL (77), the source of which was recognized after the advent of 16SrRNA sequencing. Endogenous microflora from oral cavity, vagina and gut was reported as the causative agent in 15–50% cases (80, 81). Major breakthrough to know the effect of endogenous resident vaginal flora was The Multi-Omic Microbiome Study: Pregnancy Initiative (MOMS-PI) research group, as a part of HMP-2. The study included 1,527 pregnant women with 2,06,437 samples, that included maternal vaginal, rectal, buccal, skin, and nasal swabs, urine, blood, products of conception; infant cord, cord blood, meconium, rectal, skin, and buccal swabs. All these samples were analyzed using 16S rRNA, cytokine, lipidomic profiling, metagenomics, and metatranscriptomics. It was noted that no matter with what complex or simple microbiome pregnancy was started, it eventually converged toward the well-established *Lactobacilli*-predominant microbiome by the second trimester, as has been noted by Bello previously (50).

Significant demographic variation was revealed, with a diverse vaginal microbiome and PTL being more common in African population, especially with lower socio-economic profiles. *Sneathia amnii, Prevotella-*related clades, a Lachnospiraceae taxon (BVAB1), and a *Saccharibacteria* bacterium (TM7-H1) were also noted as predictor of PTL in vaginal microbiome in these females (33, 36, 51, 82). Recently, a significant association of *Mobiluncus* and *Sneathia* with PTL has been noted by Elovitz et al. (52). An interesting correlation between Vitamin D, PTL, and vaginal microbiome has also been reported (83, 84). Vitamin D influences the vaginal microbiome by maintaining and strengthening the vaginal epithelium, by inducing expression of LL-37, an antimicrobial peptide, and most importantly, by inducing the insulin synthesis and inactivating the glycogen synthase kinase, that subsequently, stimulates the glycogen synthesis. This glycogen acts as a substrate for *Lactobacilli* and helps in lowering pH by production of lactic acid and therefore, vitamin D deficiency in a pregnant female can lead to spontaneous PTL (85–87).

Stout et al. (88) in a prospective cohort of pregnant females (2012–2015) demonstrated a much stable microbiome in pregnancies terminating into spontaneous labor as compared to pregnancies that ended up in PTL, in whom the diversity increased and stability of the microbiome decreased early in the gestation period (89). The phase of extreme variation was noted between first and second trimester with first trimester having more of the alpha diversity than others, highlighting that microbiome changes are paramount in early phases of pregnancy. However, DiGiulio et al. (81) in contrast to the other studies have shown no variation in microbiome during the different phases of pregnancy (72). Such disparities may be attributed to varied definitions of the PTL, sample size, different demographic profile, sampling techniques, analysis conventions or other confounding factors. These factors contribute to the skewing of data and don't permit a uniform analysis of results, thereby generating inconsistent inferences. However, the common conclusion of all these studies is that more diverse and lesser stable vaginal microbiome in early pregnancy correlates with PTL, thereby making early pregnancy microbial vacillations as an important marker for PTL. Though an association with *Lactobacilli*-rich vagina has been noted, but whether this association is causal or not has not been confirmed by other studies. No single taxon has been statistically correlated to PTL *per se*. This might be due to a smaller sample size; thereby necessitating need for further studies to understand the role of microbial transition between first-to-second trimesters of pregnancy. In study by DiGiulio et al. (81) weekly samples from 49 females (carried out during different trimesters and post-partum period) were integrated from vaginal as well as non-vaginal sites viz saliva, distal gut (stool), and gum or teeth. Five community state types, similar to the ones described earlier by Ravel et al. (32), along with interstate variations in vagina were noted. These variations were represented as a Markov chain and a significant correlation was observed for *Gardenella* and *Ureaplasma*, when tested independently while association with *Gardenella* remained significant on non-independent analysis. This highlights the importance of *Gardenella* in a setting of low *Lactobacilli* burden, as a potential contributor to PTL. Another significant finding noted was the association of CST IV with preterm birth, irrespective of other factors. This is in contrast to previous study by Romero et al. (90) in which the authors noted no significant difference in CST between term and preterm birth. This could be attributed to racial differences and the inclusion of gestation period of <4 weeks (90). The findings of a static microbiota are in contrast to the findings of Koren et al. (91) who noted a drastic transition in gut microbiome, termed as “remodeling” from first to third trimester and the same was seen in mice experiments also, in which the microbiota of third trimester induced adiposity along with insulin resistance in mice. This discrepancy could be attributed to the dietary modifications, use of probiotics and antibiotics by the enrolled subjects.

## Role of Microbiota in Post-Partum Period

Studies have also been conducted to note the persistence of this microbiome in pregnancy and then, later in the post-partum period. It has been shown that this *Lactobacillus*-rich microbiome falls tremendously, becoming more diverse and richer in the post-partum period, reaching the level of non-pregnant vagina, which can be attributed to varying estrogen levels (71). The post-partum microbiome tends to become similar to gut communities. The mechanism behind this correlation isn't understood entirely, while primary contrivance might be the translocation of stool microbiota to vagina. The need for this microbiota to restore itself to non-pregnant state is mandatory to have a healthy environment in case of subsequent pregnancy within a year (72). Such a shorter inter-pregnancy period might act as a stepping-stone for subsequent preterm births also, but auxiliary studies are required to corroborate this hypothesis.

## Vaginal Microbiome Other Than Bacteriome

Apart from these known bacterial communities that exist in human vagina, other microbial communities have also been traced. The major part of these communities is constituted by vaginal viral virome and very little information exists related to vaginal mycobiome. Mycobiome has revealed the role of *C.albicans* but the pathological association and relationship with other vaginal communities still needs more insight (42).

## Virome

Viral vaginal communities are one of the significant but understudied components of microbiome. Virome, in itself is an emerging concept, that encompasses all viruses residing in or on human body, be it colonizing, genome-integrated or pathogenic viruses, causing fulminant, acute, latent, chronic, or persistent infections. This human virome is composed of both prokaryotic and eukaryotic viruses, former being more common. Bacteriophages might seem to be indifferent in causing human illness, but they play a major role in causation of many infections by influencing the growth of bacterial communities (92). Similarly, endogenous retroviruses have also been implicated in many autoimmune disorders recently (93). Eukaryotic viruses, on the other hand, form a minor but a momentous component of vaginal virome with major implications in human health and disease, especially *Papillomaviruses* and *Herpesviruses* (94, 95). The study of viral genome has been possible after the advent of high-throughput sequencing and metagenomic approaches. Wylie et al. (96) was the first to conduct an in-depth study of human virome in 2012. Since then, virome at different body sites, including gut, vagina, CSF, oral cavity, conjunctiva, and cervix, has been studied. The first large-scale description of vaginal virome in human body revealed the complexity of virome at different sites viz. nose, mouth, vagina, stool, and skin. The most common viruses inhabiting the humans indeed are bacteriophages, but eukaryotic DNA viruses have also been noted from these sites, common ones being Human papilloma virus (HPV), and Herpes simplex virus (HSV). These viruses can either stipulate a defensive role to humans by their mutualistic cross-talk with bacteria or might predispose us to imminent viral infections. Further studies are required to unveil this concept of cross-talk (95). The gut virome is one of the most vast and complex ecosystems on the earth and these outnumber the bacterial cells by 10:1 and in mucus membranes with ratio of 20:1. Almost 10^8^-10^9^ virus-like particles (VLPs) are present per gram of the gut, with cross-assembly phages (crAssphage) being the dominant one. Out of this vast virome, merely ~1% has been sequenced and the major bulk is still cryptic. A transition from high bacteriophage-low bacterial community to low bacteriophage-high bacterial communities has been noted (97).

Apart from the gut virome, fewer studies have been conducted to study virome in oral cavity, CSF, cervix, respiratory system, skin, blood, conjunctiva, urinary tract, and breast milk. Skin and CSF, in absolute contrast to the gut, has low biomass communities with a predominance of bacteriophages (98, 99). The studies on virome in oral cavity have revealed the ubiquitous presence of certain genotypes, along with the personalized, persistent virome associated with some of these and is thought to be hormonal-driven. Oral virome remains stable over a period of 1 month and association of this diverse virome with periodontitis and dental plaques has been reported (100, 101). Role of virome in respiratory tract infections has also been documented but further studies are required to elucidate their pathogenic effect (14). Rodriguez et al. (13) have shown the presence of bacteriophages in 100% and HPV in 95% of the samples from urinary tract. The authors have also found some novel genotypes that accounted for carcinoma skin and were implicated in anal and genital warts too (13). Virome of conjunctiva has also been studied and it was found to have a unique paucimicrobiome with presence of *Multiple Sclerosis–associated retrovirus* (MSRV), *Human Endogenous Retrovirus K* (HERV-K), and Torque Tenovirus (TTV). Out of these, TTV has been implicated in 100% cases of seasonal hyperacute panuveitis (SHAPU) and culture-negative endophthalmitis (15). In case of cervix, *Papillomaviridae, Anelloviridae, Genomoviridae*, and *Herpesviridae* dominated different community state types (102). Asymptomatic viremia dominated by phages has been noted in different studies (11, 12). Since this virome is not static, the effect of diet, seasonal changes, vaccination, and environment on virome has been well-documented (103–105).

## Role of Vaginal Virome in Adverse Pregnancy Outcomes

Stout et al. (88) have shown that there is a specific vaginal virome in each pregnancy, which is unique to every female. The authors collected serial vaginal swabs in each triemester and subjected these to high-throughput sequencing. It was noted that there's a high probability of PTL in females with a diverse and dynamic virome rather than individuals with a consistent and static one (88). Similar findings have been observed by Wylie et al. (7, 106). Another study by Jakobsen et al. (107) revealed the abundance of ds DNA viruses in vaginal microbiome, followed by ssDNA and few unidentified viruses. The eukaryotic viruses detected were very few (4%), as the pathogenic ones are usually RNA viruses. Amongst the eukaryotic viruses isolated, *Herpesvirales*, and *Papillomaviridae* are the only pathogenic ones. A variation in bacterial vaginosis (BV) status has also been noted, revealing interplay between bacterial communities, pH and other environmental factors (107). These findings were in contrast to other studies that showed no variation in vaginal virome with BV status (108). This could be attributed to different sequencing platforms and VLP purification methods employed. Pathogenic viruses have been inversely related to presence of *Lactobacillus crispatus* in vagina. The majority is inhabited by bacteriophages, with phages enriching the vaginal flora and providing the non-host defense by forming a barrier over the mucosa. These can be lysogenic, which get integrated or are lytic which accumulate, replicate and finally, lyse the cell with release of new phages. Lytic phages have been more commonly observed in this acidic environment, in contrast to the gut virome where lysogenic phages predominate. This could be because of the fact that lysogenic phages prefer a much simpler and stable environment, in contrast to the lytic phages that inhabit a complex, more diverse, and unstable environment (109). The role of these phages in vaginal dysbiosis is multifactorial. These can either gain an entry into vaginal mucosa, colonizing the vagina, thereby replacing the commensal bacteria; or these can enter the vagina along with the pathogenic bacteria as their respective prophages, rescuing them by “superinfection exclusion” (107).

Eskew et al. (110) studied the vaginal virome in subjects undergoing *in-vitro* fertilization at the time of embryo transfer and its association with successful pregnancy and observed that a more diverse viral flora was associated with unsuccessful pregnancies and vice-versa. The association was stronger with *Papillomaviruses* and *Herpesviruses*. The authors also revealed presence of eukaryotic viruses in vaginal swabs of asymptomatic women in pre-conception period. A higher prevalence of eukaryotic pathogenic viruses in patients receiving azithromycin prophylaxis was also noted and these females eventually had an unsuccessful pregnancy outcome (110).

## Role of Vaginal Microbiota and Virome in Vertical Transmission

Newborns have feeble microbiota before birth and their gut is nearly sterile when they are born. It is the maternal microflora and the environmental influences that help an infant establish his initial microbiota (111). This flora is transmitted to infants either via maternal gut, vagina, or breast milk (112); and can occur by vertical or horizontal transfer (113–117). Amongst the initial colonizers, *Bifidobacteria* tops the list, accompanied by their respective bifidophages (43, 118). Few studies have shown that gut virome also plays an important role in shaping the microbiome of infant (119–121). Duranti et al. (122) conducted a study to profile these bifidophages along with bifidobacteria and they identified twenty-one different putative phages that were linked to *B. adolescentis, B. breve, B. dentium, B. longum*, and *B. pseudocatenulatum* (121, 123, 124). They analyzed transmission of microbiota via vertical transmission and breast-milk and found a positive correlation between the same. It has been hypothesized that bifidophages and bifidobacteria are transmitted via passage from the vagina and also via breast-milk as these have the ability to utilize human milk oligosaccharides (HMO) or HMO-derived glycan products. These also promote colonization by other commensals by helping them to co-exist by providing nutrition (44–46, 125). These authors gave the first firm evidence of maternal-to-child transmission of bifidophages (122).

## Role of Virome in Oncogenesis

It is well-established that *Lactobacillus-*dominant microbiome is beneficial in preventing dysbiosis and infections. A positive correlation between healthy vaginal milieu and viral oncogenesis has been suggested with dysbiosis playing a major role (126). Many studies have shown a protective role of *L.crispatus* in preventing HPV, HSV, and MCPyV while CST with dominance of either *L.gasseri* or *L.iners* lacked this protection and rather led to a rise in pro-inflammatory cytokines with a decrease of anti-inflammatory and anti-tumor cytokines (7, 127–130). CST with fully depleted *Lactobacilli* and dominance of *Sneathia* spp and *Prevotella* spp was seen to be associated with HPV, dysplasia and cervical carcinogenesis (131–133). However, it can't be hypothesized that vaginal microbiota is a risk factor for these viral infections, but it does influence the acquisition and clearance of such infections. There is a need for further studies to discover the basis of this “steady-state immune-bacterial crosstalk” and the interaction between bacterial microbiome and oncogenic virome.

## Limitation of the Study

The composition of microbiome is distinctive in diverse segments of vagina, however owing to dearth of this information in most of the studies discussed above, this factor couldn't be delineated in this review.

## Conclusion

All the recent studies have given an insight for vaginal microbiome, dissociating the old concept of “healthy” and “diseased,” that was mainly allied to sexually transmitted infections. These details can be used to decipher the cryptic pathogenesis of several infections and might play a major role in mucosal immunization by incorporating these beneficial microbial communities, as in probiotics. While the multi-omics have provided us with a comprehensive information regarding vaginal microbiome, but the interpretation and the translational impact on women's health still requires further understanding of functional interaction between viral communities and host cells, dynamics between different microbial communities, interaction with the host exclusively and to explore newer intervention strategies for the same. Statistical models could be used to integrate and analyze these datasets for assessing their role in biological processes, influence of environmental, and genetic factors, clinical progression, and outcome of such interactions, along with high-throughput molecular multi-omics techniques and CRISPR/Cas system. These meticulous analyses are obligatory as simple associations might represent a misinterpreted result, further confounding the scenario when translated to humans, as mere presence does not reveal any significant clinical implication on human health.

Future prospects involve the need to study evolution of virome and mycobiome in relation to their association with bacterial communities; to establish a full array of viral genes and decoding of the vaginal virome entirely, including RNA viruses, and to establish the causal association of microbiome with host under the influence of genotypic and environmental factors, using novel bioinformatic, multi-omic, statistical model, and CRISPR/Cas approaches.

## Author Contributions

All authors listed have made a substantial, direct and intellectual contribution to the work, and approved it for publication.

## Conflict of Interest

The authors declare that the research was conducted in the absence of any commercial or financial relationships that could be construed as a potential conflict of interest.

## References

[1] The NIH Human Microbiome Project (2019). Available online at: https://www.ncbi.nlm.nih.gov/pmc/articles/PMC2792171/ (accessed January 19, 2020).

[2] Lloyd-Price J Abu-Ali G Huttenhower C. The healthy human microbiome. Genome Med. (2016) 8:51. 10.1186/s13073-016-0307-y 27122046 PMC4848870

[3] A Framework for Human Microbiome Research (2019). Available online at: https://www.nature.com/articles/nature11209 (accessed January 19, 2020).

[4] Aagaard K Petrosino J Keitel W Watson M Katancik J Garcia N. The human microbiome project strategy for comprehensive sampling of the human microbiome and why it matters. FASEB J. (2013) 27:1012–22. 10.1096/fj.12-220806 23165986 PMC3574278

[5] The Human Microbiome Project Consortium. Structure, function and diversity of the healthy human microbiome. Nature. (2012). 486:207–14. 10.1038/nature11234 22699609 PMC3564958

[6] Integrative HMP (iHMP) Research Network Consortium. The Integrative Human Microbiome Project: dynamic analysis of microbiome-host omics profiles during periods of human health and disease. Cell Host Microbe. (2014). 16:276–89. 10.1016/j.chom.2014.08.014 25211071 PMC5109542

[7] Fettweis JM Serrano MG Brooks JP Edwards DJ Girerd PH Parikh HI. The vaginal microbiome and preterm birth. Nat Med. (2019) 25:1012–21. 10.1038/s41591-019-0450-2 31142849 PMC6750801

[8] Lloyd-Price J Arze C Ananthakrishnan AN Schirmer M Avila-Pacheco J Poon TW. Multi-omics of the gut microbial ecosystem in inflammatory bowel diseases. Nature. (2019) 569:655–62. 10.1038/s41586-019-1237-9 31142855 PMC6650278

[9] Zhou W Sailani MR Contrepois K Zhou Y Ahadi S Leopold SR. Longitudinal multi-omics of host–microbe dynamics in prediabetes. Nature. (2019) 569:663–71. 10.1038/s41586-019-1236-x 31142858 PMC6666404

[10] Human Gut Microbiome: Hopes Threats and Promises (2019). Available online at: https://gut.bmj.com/content/67/9/1716 (accessed January 19, 2020).

[11] Sauvage V Laperche S Cheval J Muth E Dubois M Boizeau L. Viral metagenomics applied to blood donors and recipients at high risk for blood-borne infections. Blood Transfus. (2016) 14:400–7. 10.2450/2016.0160-15 27136432 PMC5016298

[12] Moustafa A Xie C Kirkness E Biggs W Wong E Turpaz Y. The blood DNA virome in 8,000 humans. PLoS Pathog. (2017) 13:e1006292. 10.1371/journal.ppat.1006292 28328962 PMC5378407

[13] Santiago-Rodriguez TM Ly M Bonilla N Pride DT. The human urine virome in association with urinary tract infections. Front Microbiol. (2015) 6:14. 10.3389/fmicb.2015.00014 25667584 PMC4304238

[14] Wylie KM. The virome of the human respiratory tract. Clin Chest Med. (2017) 38:11–9. 10.1016/j.ccm.2016.11.001 28159153 PMC7115714

[15] Doan T Akileswaran L Andersen D Johnson B Ko N Shrestha A. Paucibacterial microbiome and resident DNA virome of the healthy conjunctiva. Invest Ophthalmol Vis Sci. (2016) 57:5116–26. 10.1167/iovs.16-19803 27699405 PMC5054734

[16] Kim JM Park YJ Lactobacillus and urine microbiome in association with urinary tract infections and bacterial vaginosis. Urogenital Tract Infection. (2018) 13:7–13. 10.14777/uti.2018.13.1.7

[17] Is There a Protective Role for Vaginal Flora? (2019) Available online at: https://pubmed.ncbi.nlm.nih.gov/11095812-is-there-a-protective-role-for-vaginal-flora/ (accessed January 19, 2020).

[18] Intravaginal Practices Vaginal Flora Disturbances and Acquisition of Sexually Transmitted Diseases in Zimbabwean Women (2019). Available online at: https://pubmed.ncbi.nlm.nih.gov/10669342-intravaginal-practices-vaginal-flora-disturbances-and-acquisition-of-sexually-transmitted-diseases-in-zimbabwean-women/ (accessed January 19, 2020). 10.1086/315227 10669342

[19] Amann RI Ludwig W Schleifer KH. Phylogenetic identification and in situ detection of individual microbial cells without cultivation. Microbiol Rev. (1995) 59:143–69. 10.1128/MMBR.59.1.143-169.1995 7535888 PMC239358

[20] Hugenholtz P Goebel BM Pace NR. Impact of culture-independent studies on the emerging phylogenetic view of bacterial diversity. J Bacteriol. (1998) 180:4765–74. 10.1128/JB.180.18.4765-4774.1998 9733676 PMC107498

[21] Separation and Purification of Bacteria From Soil (2019). Available online at: https://aem.asm.org/content/49/6/1482 (accessed January 19, 2020).

[22] Boskey ER Telsch KM Whaley KJ Moench TR Cone RA. Acid production by vaginal flora in vitro is consistent with the rate and extent of vaginal acidification. Infect Immun. (1999) 67:5170–5. 10.1128/IAI.67.10.5170-5175.1999 10496892 PMC96867

[23] Antimicrobial Activity and Characteristics of Bacteriocins Produced by Vaginal Lactobacilli (2019). Available online at: https://www.researchgate.net/publication/289185215_Antimicrobial_activity_and_characteristics_of_bacteriocins_produced_by_vaginal_Lactobacilli (accessed January 19, 2020).

[24] Defense Factors of Vaginal Lactobacilli (2019). Available online at: https://pubmed.ncbi.nlm.nih.gov/11518895/ (accessed January 19, 2020).

[25] Hawes SE Hillier SL Benedetti J Stevens CE Koutsky LA Wolner-Hanssen P. Hydrogen peroxide-producing lactobacilli and acquisition of vaginal infections. J Infect Dis. (1996) 174:1058–63. 10.1093/infdis/174.5.1058 8896509

[26] Prevalence of Hydrogen Peroxide-Producing Lactobacillus Species in Normal Women and Women With Bacterial Vaginosis (2019). Available online at: https://pubmed.ncbi.nlm.nih.gov/2915019-prevalence-of-hydrogen-peroxide-producing-lactobacillus-species-in-normal-women-and-women-with-bacterial-vaginosis/ (accessed January 19, 2020). 10.1128/jcm.27.2.251-256.1989 PMC267286 2915019

[27] Witkin SS Alvi S Bongiovanni AM Linhares IM Ledger WJ. Lactic acid stimulates interleukin-23 production by peripheral blood mononuclear cells exposed to bacterial lipopolysaccharide. FEMS Immunol Med Microbiol. (2011) 61:153–8. 10.1111/j.1574-695X.2010.00757.x 21118312

[28] Novel Vaginal Microflora Colonization Model Providing New Insight Into Microbicide Mechanism of Action (2019). Available online at: https://pubmed.ncbi.nlm.nih.gov/22027006-novel-vaginal-microflora-colonization-model-providing-new-insight-into-microbicide-mechanism-of-action/ (accessed January 19, 2020). 10.1128/mBio.00168-11 PMC3202752 22027006

[29] The Identification of Vaginal Lactobacillus Species and the Demographic and Microbiologic Characteristics of Women Colonized by These Species (2019). Available online at: https://pubmed.ncbi.nlm.nih.gov/10558952-the-identification-of-vaginal-lactobacillus-species-and-the-demographic-and-microbiologic-characteristics-of-women-colonized-by-these-species/ (accessed January 19, 2020). 10.1086/315109 10558952

[30] Phenotypic and Phylogenetic Characterization of a Novel Lactobacillus Species From Human Sources: Description of Lactobacillus Iners sp. nov (2019). Available online at: https://www.microbiologyresearch.org/content/journal/ijsem/10.1099/00207713-49-1-217 (accessed January 19, 2020). 10.1099/00207713-49-1-217 10028266

[31] Characterization of Vaginal Microbial Communities in Adult Healthy Women Using Cultivation-Independent Methods (2019). Available online at: https://pubmed.ncbi.nlm.nih.gov/15289553-characterization-of-vaginal-microbial-communities-in-adult-healthy-women-using-cultivation-independent-methods/ (accessed January 19, 2020). 10.1099/mic.0.26905-0 15289553

[32] Vaginal Microbiome of Reproductive-Age Women. (2019). Available online at: https://pubmed.ncbi.nlm.nih.gov/20534435-vaginal-microbiome-of-reproductive-age-women/ (accessed January 19, 2020).

[33] Replication Refinement of a Vaginal Microbial Signature of Preterm Birth in Two Racially Distinct Cohorts of US Women. (2019). Available online at: https://pubmed.ncbi.nlm.nih.gov/28847941-replication-and-refinement-of-a-vaginal-microbial-signature-of-preterm-birth-in-two-racially-distinct-cohorts-of-us-women/ (accessed January 19, 2020).

[34] Santiago GLDS Cools P Verstraelen H Trog M Missine G El Aila N. Longitudinal study of the dynamics of vaginal microflora during two consecutive menstrual cycles. PLoS ONE. (2011) 6:e28180. 10.1371/journal.pone.0028180 22140538 PMC3227645

[35] Verstraelen H Verhelst R Claeys G De Backer E Temmerman M Vaneechoutte M. Longitudinal analysis of the vaginal microflora in pregnancy suggests that L. crispatus promotes the stability of the normal vaginal microflora and that L. gasseri and/or L. iners are more conducive to the occurrence of abnormal vaginal microflora. BMC Microbiol. (2009) 9:116. 10.1186/1471-2180-9-116 19490622 PMC2698831

[36] Kindinger LM Bennett PR Lee YS Marchesi JR Smith A Cacciatore S. The interaction between vaginal microbiota, cervical length, and vaginal progesterone treatment for preterm birth risk. Microbiome. (2017) 5:6. 10.1186/s40168-016-0223-9 28103952 PMC5244550

[37] Differences in the Composition of Vaginal Microbial Communities Found in Healthy Caucasian Black Women. (2019). Available online at: https://pubmed.ncbi.nlm.nih.gov/18043622-differences-in-the-composition-of-vaginal-microbial-communities-found-in-healthy-caucasian-and-black-women/ (accessed January 19, 2020).

[38] Mania-Pramanik J Kerkar SC Mehta PB Potdar S Salvi VS. Use of vaginal pH in diagnosis of infections and its association with reproductive manifestations. J Clin Lab Anal. (2008) 22:375–9. 10.1002/jcla.20273 18803273 PMC6649105

[39] Zhou X Hansmann MA Davis CC Suzuki H Brown CJ Schütte U. The vaginal bacterial communities of Japanese women resemble those of women in other racial groups. FEMS Immunol Med Microbiol. (2010) 58:169–81. 10.1111/j.1574-695X.2009.00618.x 19912342 PMC2868947

[40] Microbes on the Human Vaginal Epithelium. (2019). Available online at: https://pubmed.ncbi.nlm.nih.gov/15911771-microbes-on-the-human-vaginal-epithelium/ (accessed January 19, 2020).

[41] Forney LJ Foster JA Ledger W. The vaginal flora of healthy women is not always dominated by Lactobacillus species. J Infect Dis. (2006) 194:1468–9; author reply 1469–70. 10.1086/508497 17054080

[42] Bradford LL Ravel J. The vaginal mycobiome: A contemporary perspective on fungi in women's health and diseases. Virulence. (2017) 8:342–51. 10.1080/21505594.2016.1237332 27657355 PMC5411243

[43] Duranti S Gaiani F Mancabelli L Milani C Grandi A Bolchi A. Elucidating the gut microbiome of ulcerative colitis: bifidobacteria as novel microbial biomarkers. FEMS Microbiol Ecol. (2016) 92:fiw191. 10.1093/femsec/fiw191 27604252

[44] Egan M O'Connell Motherway M Kilcoyne M Kane M Joshi L Ventura M. Cross-feeding by Bifidobacterium breve UCC2003 during co-cultivation with Bifidobacterium bifidum PRL2010 in a mucin-based medium. BMC Microbiol. (2014) 14:282. 10.1186/s12866-014-0282-7 25420416 PMC4252021

[45] Metabolism of Sialic Acid by Bifidobacterium Breve UCC2003. (2020). Available online at: https://aem.asm.org/content/80/14/4414 (accessed January 19, 2020).

[46] Turroni F Özcan E Milani C Mancabelli L Viappiani A van Sinderen D. Glycan cross-feeding activities between bifidobacteria under in vitro conditions. Front Microbiol. (2015) 6:1030. 10.3389/fmicb.2015.01030 26441950 PMC4585166

[47] Relman DA. “Til death do us part”: coming to terms with symbiotic relationships. Forward. Nat Rev Microbiol. (2008). 6:721–4. 10.1038/nrmicro1990 19086265

[48] Evolution of Mammals Their Gut Microbes. (2019). Available online at: https://pubmed.ncbi.nlm.nih.gov/18497261-evolution-of-mammals-and-their-gut-microbes/ (accessed January 19, 2020).

[49] Turnbaugh PJ Ley RE Hamady M Fraser-Liggett CM Knight R Gordon JI. The human microbiome project. Nature. (2007) 449:804–10. 10.1038/nature06244 17943116 PMC3709439

[50] Dominguez-Bello MG. Gestational shaping of the maternal vaginal microbiome. Nat Med. (2019) 25:882–3. 10.1038/s41591-019-0483-6 31142848

[51] Serrano MG Parikh HI Brooks JP Edwards DJ Arodz TJ Edupuganti L. Racioethnic diversity in the dynamics of the vaginal microbiome during pregnancy. Nat Med. (2019) 25:1001–11. 10.1038/s41591-019-0465-8 31142850 PMC6746180

[52] Elovitz MA Gajer P Riis V Brown AG Humphrys MS Holm JB. Cervicovaginal microbiota and local immune response modulate the risk of spontaneous preterm delivery. Nat Commun. (2019) 10:1–8. 10.1038/s41467-019-09285-9 30899005 PMC6428888

[53] Carda-Diéguez M Cárdenas N Aparicio M Beltrán D Rodríguez JM Mira A Variations in vaginal, penile, and oral microbiota after sexual intercourse: a case report. Front Med. (2019) 6:178 10.3389/fmed.2019.00294 PMC6692966 31440511

[54] Tejesvi MV Nissi R Saravesi K Pirttilä AM Markkola A Talvensaari-Mattila A. Association of prevalent vaginal microbiome of mother with occurrence of type I diabetes in child. Sci Rep. (2019) 9:1–6. 10.1038/s41598-018-37467-w 30700742 PMC6353987

[55] Kolde R Franzosa EA Rahnavard G Hall AB Vlamakis H Stevens C. Host genetic variation and its microbiome interactions within the human microbiome project. Genome Med. (2018) 10:6. 10.1186/s13073-018-0515-8 29378630 PMC5789541

[56] Stinson LF Payne MS Keelan JA. A critical review of the bacterial baptism hypothesis and the impact of cesarean delivery on the infant microbiome. Front Med. (2018) 5:135. 10.3389/fmed.2018.00135 29780807 PMC5945806

[57] Neu J Rushing J. Cesarean versus vaginal delivery: long term infant outcomes and the hygiene hypothesis. Clin Perinatol. (2011) 38:321–31. 10.1016/j.clp.2011.03.008 21645799 PMC3110651

[58] Klebanoff MA Schwebke JR Zhang J Nansel TR Yu KF Andrews WW. Vulvovaginal symptoms in women with bacterial vaginosis. Obstet Gynecol. (2004) 104:267–72. 10.1097/01.AOG.0000134783.98382.b0 15291998

[59] Screening for Bacterial Vaginosis in Pregnancy to Prevent Preterm Delivery - U,.S. Preventive Services Task Force - American Family Physician (2019) Available online at: https://www.aafp.org/afp/2008/0701/p106.html (accessed January 19, 2020).

[60] Brotman RM Ravel J Cone RA Zenilman JM. Rapid fluctuation of the vaginal microbiota measured by Gram stain analysis. Sex Transm Infect. (2010) 86:297–302. 10.1136/sti.2009.040592 20660593 PMC3534767

[61] Hay PE Ugwumadu A Chowns J. Sex, thrush and bacterial vaginosis. Int J STD AIDS. (1997) 8:603–8. 10.1258/0956462971918850 9310218

[62] What's New in Bacterial Vaginosis and Trichomoniasis? (2019) Available online at: https://pubmed.ncbi.nlm.nih.gov/15963878-whats-new-in-bacterial-vaginosis-and-trichomoniasis/ (accessed January 19, 2020).

[63] Keane FE Ison CA Taylor-Robinson D. A longitudinal study of the vaginal flora over a menstrual cycle. Int J STD AIDS. (1997) 8:489–94. 10.1258/0956462971920631 9259496

[64] Intrauterine Infection Prematurity. (2020). Available online at: https://pubmed.ncbi.nlm.nih.gov/11921380-intrauterine-infection-and-prematurity/ (accessed January 19, 2020).

[65] Tulchinsky D Hobel CJ Yeager E Marshall JR. Plasma estrone, estradiol, estriol, progesterone, and 17-hydroxyprogesterone in human pregnancy. Am J Obstet Gynecol. (2019) 112:1095–100. 10.1016/0002-9378(72)90185-8 5025870

[66] REVIEW ARTICLE: The Immune System in Pregnancy: A Unique Complexity - Mor – 2010 - American Journal of Reproductive Immunology - Wiley Online Library. (2019). Available online at: https://onlinelibrary.wiley.com/doi/full/10.1111/j.1600-0897.2010.00836.x (accessed January 19, 2020). 10.1111/j.1600-0897.2010.00836.x PMC3025805 20367629

[67] Relationships Between Mechanical Properties Extracellular Matrix Constituents of the Cervical Stroma During Pregnancy. (2019). Available online at: https://www.sciencedirect.com/science/article/abs/pii/S0146000509000421?via%3Dihub (accessed January 19, 2020). 10.1053/j.semperi.2009.06.002 PMC2774809 19796726

[68] A Metagenomic Approach to Characterization of the Vaginal Microbiome Signature in Pregnancy. (2019). Available online at: https://journals.plos.org/plosone/article?id=10.1371/journal.pone.0036466 (accessed January 19, 2020). 10.1371/journal.pone.0036466 PMC3374618 22719832

[69] The Composition Stability of the Vaginal Microbiota of Normal Pregnant Women Is Different From That of Non-Pregnant Women. (2019). Available online at: https://microbiomejournal.biomedcentral.com/articles/10.1186/2049-2618-2-4 (accessed January 19, 2020).

[70] Pregnancy's Stronghold on the Vaginal Microbiome. (2019). Available online at: https://journals.plos.org/plosone/article?id=10.1371/journal.pone.0098514 (accessed January 19, 2020).

[71] The Vaginal Microbiome During Pregnancy the Postpartum Period in a European Population. (2019). Available online at: https://www.nature.com/articles/srep08988 (accessed January 19, 2020).

[72] Temporal Spatial Variation of the Human Microbiota During Pregnancy. (2019). 10.1073/pnas.1502875112 Available online at: https://www.pnas.org/content/112/35/11060 (accessed January 19, 2020).

[73] Freitas AC Chaban B Bocking A Rocco M Yang S Hill JE. The vaginal microbiome of pregnant women is less rich and diverse, with lower prevalence of Mollicutes, compared to non-pregnant women. Sci Rep. (2017) 7:9212. 10.1038/s41598-017-07790-9 28835692 PMC5569030

[74] Devillard E Burton JP Hammond JA Lam D Reid G. Novel insight into the vaginal microflora in postmenopausal women under hormone replacement therapy as analyzed by PCR-denaturing gradient gel electrophoresis. Eur J Obstet Gynecol Reprod Biol. (2004) 117:76–81. 10.1016/j.ejogrb.2004.02.001 15474249

[75] Farage MA Miller KW Sobel JD Dynamics of the vaginal ecosystem—hormonal influences. Infect Dis. (2010) 3:IDRT.S3903 10.4137/IDRT.S3903

[76] Liu L Oza S Hogan D Chu Y Perin J Zhu J. Global, regional, and national causes of under-5 mortality in 2000-15: an updated systematic analysis with implications for the Sustainable Development Goals. Lancet. (2016) 388:3027–35. 10.1016/S0140-6736(16)31593-8 27839855 PMC5161777

[77] Romero R Dey SK Fisher SJ. Preterm labor: one syndrome, many causes. Science. (2014) 345:760–5. 10.1126/science.1251816 25124429 PMC4191866

[78] Mor G Aldo P Alvero AB. The unique immunological and microbial aspects of pregnancy. Nat Rev Immunol. (2017) 17:469–82. 10.1038/nri.2017.64 28627518

[79] Jefferson KK Chapter One - the bacterial etiology of preterm birth. In: Sariaslani S, Gadd GM, editors. Advances in Applied Microbiology. Academic Press (2012). 80:1–22. 10.1016/B978-0-12-394381-1.00001-5 22794142

[80] Microbial Prevalence Diversity Abundance in Amniotic Fluid During Preterm Labor: A Molecular Culture-Based Investigation. (2019). Available online at: https://pubmed.ncbi.nlm.nih.gov/18725970-microbial-prevalence-diversity-and-abundance-in-amniotic-fluid-during-preterm-labor-a-molecular-and-culture-based-investigation/ (accessed January 19, 2020). 10.1371/journal.pone.0003056 PMC2516597 18725970

[81] DiGiulio DB Romero R Kusanovic JP Gómez R Kim CJ Seok KS. Prevalence and diversity of microbes in the amniotic fluid, the fetal inflammatory response, and pregnancy outcome in women with preterm pre-labor rupture of membranes. Am J Reprod Immunol. (2010) 64:38–57. 10.1111/j.1600-0897.2010.00830.x 20331587 PMC2907911

[82] Brown RG Marchesi JR Lee YS Smith A Lehne B Kindinger LM. Vaginal dysbiosis increases risk of preterm fetal membrane rupture, neonatal sepsis and is exacerbated by erythromycin. BMC Med. (2018) 16:9. 10.1186/s12916-017-0999-x 29361936 PMC5782380

[83] Jefferson KK Parikh HI Garcia EM Edwards DJ Serrano MG Hewison M. Relationship between vitamin D status and the vaginal microbiome during pregnancy. J Perinatol. (2019) 39:824–36. 10.1038/s41372-019-0343-8 30858609 PMC6535112

[84] Zhou SS Tao YH Huang K Zhu BB Tao FB. Vitamin D and risk of preterm birth: Up-to-date meta-analysis of randomized controlled trials and observational studies. J Obstet Gynaecol Res. (2017) 43:247–56. 10.1111/jog.13239 28150405

[85] Maestro B Molero S Bajo S Dávila N Calle C. Transcriptional activation of the human insulin receptor gene by 1,25-dihydroxyvitamin D(3). Cell Biochem Funct. (2002) 20:227–32. 10.1002/cbf.951 12125099

[86] Stimulation by 1 25-dihydroxyvitamin D3 of Insulin Receptor Expression Insulin Responsiveness for Glucose Transport in U-937 Human Promonocytic Cells. (2019). Available online at: https://pubmed.ncbi.nlm.nih.gov/11075718-stimulation-by-125-dihydroxyvitamin-d3-of-insulin-receptor-expression-and-insulin-responsiveness-for-glucose-transport-in-u-937-human-promonocytic-cells/ (accessed January 19, 2020). 10.1507/endocrj.47.383 11075718

[87] Parker L Levinger I Mousa A Howlett K de Courten B. Plasma 25-Hydroxyvitamin D is related to protein signaling involved in glucose homeostasis in a tissue-specific manner. Nutrients. (2016) 8:631. 10.3390/nu8100631 27754361 PMC5084018

[88] Stout MJ Tuuli MG Macones GA Wylie TN Wylie KM 30: Diversity of the vaginal virome is associated with preterm birth. Am J Obstet Gynecol. (2018). 218:S23 10.1016/j.ajog.2017.10.441 PMC6066425 29738749

[89] Early Pregnancy Vaginal Microbiome Trends Preterm Birth. (2019). Available online at: https://pubmed.ncbi.nlm.nih.gov/28549981-early-pregnancy-vaginal-microbiome-trends-and-preterm-birth/ (accessed January 19, 2020).

[90] Romero R Hassan SS Gajer P Tarca AL Fadrosh DW Bieda J. The vaginal microbiota of pregnant women who subsequently have spontaneous preterm labor and delivery and those with a normal delivery at term. Microbiome. (2014) 2:18. 10.1186/2049-2618-2-18 24987521 PMC4066267

[91] Koren O Goodrich JK Cullender TC Spor A Laitinen K Bäckhed HK. Host remodeling of the gut microbiome and metabolic changes during pregnancy. Cell. (2012) 150:470–80. 10.1016/j.cell.2012.07.008 22863002 PMC3505857

[92] Waldor MK Mekalanos JJ. Lysogenic conversion by a filamentous phage encoding cholera toxin. Science. (1996) 272:1910–4. 10.1126/science.272.5270.1910 8658163

[93] Implication of Human Endogenous Retroviruses in the Development of Autoimmune Diseases. (2020). Available online at: https://pubmed.ncbi.nlm.nih.gov/20635879/ (accessed January 19, 2020).

[94] Peterson J Garges S Giovanni M McInnes P Wang L Schloss JA. The NIH human microbiome project. Genome Res. (2009) 19:2317–23. 10.1101/gr.096651.109 19819907 PMC2792171

[95] Wylie KM Mihindukulasuriya KA Zhou Y Sodergren E Storch GA Weinstock GM. Metagenomic analysis of double-stranded DNA viruses in healthy adults. BMC Biol. (2014) 12:71. 10.1186/s12915-014-0071-7 25212266 PMC4177058

[96] Wylie KM Weinstock GM Storch GA. Emerging view of the human virome. Transl Res. (2012) 160:283–90. 10.1016/j.trsl.2012.03.006 22683423 PMC3701101

[97] Mukhopadhya I Segal JP Carding SR Hart AL Hold GL. The gut virome: the ‘missing link' between gut bacteria and host immunity? Therap Adv Gastroenterol. (2019) 12:1756284819836620. 10.1177/1756284819836620 30936943 PMC6435874

[98] Hannigan GD Meisel JS Tyldsley AS Zheng Q Hodkinson BP SanMiguel AJ. The human skin double-stranded DNA virome: topographical and temporal diversity, genetic enrichment, and dynamic associations with the host microbiome. MBio. (2015) 6:e01578–15. 10.1128/mBio.01578-15 26489866 PMC4620475

[99] Ghose C Ly M Schwanemann LK Shin JH Atab K Barr JJ. The virome of cerebrospinal fluid: viruses where we once thought there were none. Front Microbiol. (2019) 10:2061. 10.3389/fmicb.2019.02061 31555247 PMC6742758

[100] Abeles SR Robles-Sikisaka R Ly M Lum AG Salzman J Boehm TK. Human oral viruses are personal, persistent and gender-consistent. ISME J. (2014) 8:1753–67. 10.1038/ismej.2014.31 24646696 PMC4139723

[101] Ly M Abeles SR Boehm TK Robles-Sikisaka R Naidu M Santiago-Rodriguez T. Altered oral viral ecology in association with periodontal disease. MBio. (2014) 5:e01133–14. 10.1128/mBio.01133-14 24846382 PMC4030452

[102] Siqueira JD Curty G Xutao D Hofer CB Machado ES Seuánez HN. Composite analysis of the virome and bacteriome of HIV/HPV co-infected women reveals proxies for immunodeficiency. Viruses. (2019) 11:422. 10.3390/v11050422 31067713 PMC6563245

[103] Jamieson AM. Influence of the microbiome on response to vaccination. Hum Vaccin Immunother. (2015) 11:2329–31. 10.1080/21645515.2015.1022699 26090701 PMC4635895

[104] Minot S Sinha R Chen J Li H Keilbaugh SA Wu GD. The human gut virome: inter-individual variation and dynamic response to diet. Genome Res. (2011) 21:1616–25. 10.1101/gr.122705.111 21880779 PMC3202279

[105] Prussin AJ Torres PJ Shimashita J Head SR Bibby KJ Kelley ST. Seasonal dynamics of DNA and RNA viral bioaerosol communities in a daycare center. Microbiome. (2019) 7:53. 10.1186/s40168-019-0672-z 30935423 PMC6444849

[106] Wylie KM Wylie TN Cahill AG Macones GA Tuuli MG Stout MJ. The vaginal eukaryotic DNA virome and preterm birth. Am J Obstet Gynecol. (2018) 219:189.e1–12. 10.1016/j.ajog.2018.04.048 29738749 PMC6066425

[107] Jakobsen RR Haahr T Humaidan P Jensen JS Kot W Castro-Mejia J Characterization of the vaginal DNA virome in health and dysbiosis: an opening study in patients with non-female factor infertility. bioRxiv. (2019) 7 10.1101/755710 PMC7600586 33050261

[108] Gosmann C Anahtar MN Handley SA Farcasanu M Abu-Ali G Bowman BA. Lactobacillus -deficient cervicovaginal bacterial communities are associated with increased hiv acquisition in young South African women. Immunity. (2017) 46:29–37. 10.1016/j.immuni.2016.12.013 28087240 PMC5270628

[109] Well-Temperate Phage: Optimal Bet-Hedging Against Local Environmental Collapses (2020). Available online at: https://www.nature.com/articles/srep10523 (accessed January 19, 2020). 10.1038/srep10523 PMC4451807 26035282

[110] Eskew AM Stout MJ Bedrick BS Riley JK Omurtag KR Jimenez PT Association of the eukaryotic vaginal virome with prophylactic antibiotic exposure and reproductive outcomes in a subfertile population undergoing in vitro fertilisation: a prospective exploratory study. BJOG Int J Obstet Gynaecol. (2019) 127:208–16. 10.1111/1471-0528.15951 PMC6910962 31529767

[111] Sim K Powell E Shaw AG McClure Z Bangham M Kroll JS. The neonatal gastrointestinal microbiota: the foundation of future health? Arch Dis Child Fetal Neonatal Ed. (2013) 98:F362–4. 10.1136/archdischild-2012-302872 23221466

[112] The Influence of Early Infant-Feeding Practices on the Intestinal Microbiome Body Composition in Infants. (2020). Available online at: https://pubmed.ncbi.nlm.nih.gov/26715853-the-influence-of-early-infant-feeding-practices-on-the-intestinal-microbiome-and-body-composition-in-infants/ (accessed January 19, 2020).

[113] Azad MB Konya T Maughan H Guttman DS Field CJ Chari RS. Gut microbiota of healthy Canadian infants: profiles by mode of delivery and infant diet at 4 months. CMAJ. (2013) 185:385–94. 10.1503/cmaj.121189 23401405 PMC3602254

[114] Rosa PSL Warner BB Zhou Y Weinstock GM Sodergren E Hall-Moore CM. Patterned progression of bacterial populations in the premature infant gut. PNAS. (2014) 111:12522–7. 10.1073/pnas.1409497111 25114261 PMC4151715

[115] Dominguez-Bello MG Costello EK Contreras M Magris M Hidalgo G Fierer N. Delivery mode shapes the acquisition and structure of the initial microbiota across multiple body habitats in newborns. PNAS. (2010) 107:11971–5. 10.1073/pnas.1002601107 20566857 PMC2900693

[116] Bäckhed F Ley RE Sonnenburg JL Peterson DA Gordon JI. Host-Bacterial Mutualism in the Human Intestine. Science. (2005) 307:1915–20. 10.1126/science.1104816 15790844

[117] Zhang C Derrien M Levenez F Brazeilles R Ballal SA Kim J. Ecological robustness of the gut microbiota in response to ingestion of transient food-borne microbes. ISME J. (2016) 10:2235–45. 10.1038/ismej.2016.13 26953599 PMC4989305

[118] Turroni F Taverniti V Ruas-Madiedo P Duranti S Guglielmetti S Lugli GA. Bifidobacterium bifidum PRL2010 modulates the host innate immune response. Appl Environ Microbiol. (2014) 80:730–40. 10.1128/AEM.03313-13 24242237 PMC3911076

[119] Lim ES Zhou Y Zhao G Bauer IK Droit L Ndao IM. Early life dynamics of the human gut virome and bacterial microbiome in infants. Nat Med. (2015) 21:1228–34. 10.1038/nm.3950 26366711 PMC4710368

[120] Gut Virome Sequencing in Children With Early Islet Autoimmunity (2020). Available online at: https://care.diabetesjournals.org/content/38/5/930 (accessed January 19, 2020).

[121] Prophages of the Genus Bifidobacterium as Modulating Agents of the Infant Gut Microbiota - Lugli – 2016 - Environmental Microbiology - Wiley Online Library. (2020). Available online at: https://sfamjournals.onlinelibrary.wiley.com/doi/abs/10.1111/1462-2920.13154 (accessed January 19, 2020). 10.1111/1462-2920.13154 26627180

[122] Duranti S Lugli GA Mancabelli L Armanini F Turroni F James K. Maternal inheritance of bifidobacterial communities and bifidophages in infants through vertical transmission. Microbiome. (2017) 5:66. 10.1186/s40168-017-0282-6 28651630 PMC5485682

[123] Comparative Analyses of Prophage-Like Elements Present in Bifidobacterial Genomes. (2020). Available online at: https://aem.asm.org/content/75/21/6929 (accessed January 19, 2020). 10.1128/AEM.01112-09 PMC2772436 19734330

[124] Prophage-Like Elements in Bifidobacteria: Insights From Genomics Transcription Integration Distribution Phylogenetic Analysis. (2020). Available online at: https://aem.asm.org/content/71/12/8692 (accessed January 19, 2020). 10.1128/AEM.71.12.8692-8705.2005 PMC1317369 16332864

[125] Deciphering Bifidobacterial-Mediated Metabolic Interactions Their Impact on Gut Microbiota by a Multi-Omics Approach. (2020). Available online at: https://www.nature.com/articles/ismej2015236 (accessed January 19, 2020). 10.1038/ismej.2015.236 PMC4918443 26859770

[126] Vaginal Dysbiosis the Risk of Human Papillomavirus Cervical Cancer: Systematic Review Meta-Analysis. (2020). Available online at: https://www.ajog.org/article/S0002-9378(18)32221-X/fulltext (accessed January 19, 2020).

[127] Mousavi E Makvandi M Teimoori A Ataei A Ghafari S Samarbaf-Zadeh A. Antiviral effects of Lactobacillus crispatus against HSV-2 in mammalian cell lines. J Chin Med Assoc. (2018) 81:262–7. 10.1016/j.jcma.2017.07.010 29037754

[128] The Association of Uterine Cervical Microbiota With an Increased Risk for Cervical Intraepithelial Neoplasia in Korea. (2020). Available online at: https://www.clinicalmicrobiologyandinfection.com/article/S1198-743X(15)00318-3/fulltext (accessed January 19, 2020). 10.1016/j.cmi.2015.02.026 25752224

[129] Zanotta N Delbue S Signorini L Villani S D'Alessandro S Campisciano G. Merkel cell polyomavirus is associated with anal infections in men who have sex with men. Microorganisms. (2019) 7:54. 10.3390/microorganisms7020054 30791443 PMC6406607

[130] Yang X Da M Zhang W Qi Q Zhang C Han S Role of <em>Lactobacillus</em> in cervical cancer. Cancer Manag Res. (2018) 10:1219–29. 10.2147/CMAR.S165228 29844701 PMC5962305

[131] Łaniewski P Barnes D Goulder A Cui H Roe DJ Chase DM. Linking cervicovaginal immune signatures, HPV and microbiota composition in cervical carcinogenesis in non-Hispanic and Hispanic women. Sci Rep. (2018) 8:7593. 10.1038/s41598-018-25879-7 29765068 PMC5954126

[132] Mitra A MacIntyre DA Lee YS Smith A Marchesi JR Lehne B. Cervical intraepithelial neoplasia disease progression is associated with increased vaginal microbiome diversity. Sci Rep. (2015) 5:16865. 10.1038/srep16865 26574055 PMC4648063

[133] Association of the Vaginal Microbiota with Human Papillomavirus Infection in a Korean Twin Cohort. (2020). Available online at: https://journals.plos.org/plosone/article?id=10.1371/journal.pone.0063514 (accessed January 19, 2020). 10.1371/journal.pone.0063514 PMC3661536 23717441

